# Influence of IL-β, IL-1RN, and TNF-α variants on the risk of acetylsalicylic acid-induced upper gastrointestinal bleeding: a case-control study

**DOI:** 10.31744/einstein_journal/2024AO0746

**Published:** 2024-08-12

**Authors:** Marcela Forgerini, Cleslei Fernando Zanelli, Sandro Roberto Valentini, Patrícia de Carvalho Mastroianni

**Affiliations:** 1 Faculdade de Ciências Farmacêuticas Universidade Estadual Paulista “Júlio de Mesquita Filho” Araraquara SP Brazil Faculdade de Ciências Farmacêuticas, Universidade Estadual Paulista “Júlio de Mesquita Filho”, Araraquara, SP, Brazil.

**Keywords:** Drug-related side effects and adverse reactions, Interleukins, Pharmacogenetics, Platelet aggregation inhibitors, Tumor necrosis factor-alpha

## Abstract

**Objective:**

The use of acetylsalicylic acid, even in low doses, may be associated with the onset of upper gastrointestinal bleeding as an idiosyncratic response. Considering the role of the genetic background in inter-individual responses to pharmacotherapy, we aimed to investigate the role of seven variants in the *TNF-*α, *IL-*β, and *IL-1RN* genes in association with the risk of upper gastrointestinal bleeding in users of low-dose acetylsalicylic acid for the prevention of cardiovascular events.

**Methods:**

A case-control study was conducted in a Brazilian hospital complex. The Case Group comprised patients diagnosed with upper gastrointestinal bleeding who were administered a low dose of acetylsalicylic acid (n=50). Two Control Groups were recruited: 1) low-dose acetylsalicylic acid users without gastrointestinal complaints and under the supervision of a cardiologist (n=50) and 2) healthy controls (n=189). Sociodemographic, clinical, pharmacotherapeutic, and lifestyle data were recorded through face-to-face interviews. Genomic DNA from all participants was genotyped for rs16944 and rs1143634 (*IL-*β gene), rs4251961 (*IL-1RN* gene), and rs1799964, rs1799724, rs361525, and rs1800629 (*TNF-*α gene).

**Results:**

No significant difference was noted in the genotypic frequencies of TNF-α, IL-β, and IL-1RN variants between the Case and Control Groups of low-dose acetylsalicylic acid users (p>0.05). The frequency of rs1800629 genotypes (*TNF-*α gene) differed significantly between the Case Group and healthy controls (p=0.003). None of the evaluated variants were associated with a risk of upper gastrointestinal bleeding.

**Conclusion:**

This study aimed to explore pharmacogenomics biomarkers in low-dose acetylsalicylic acid users. Our data suggest that the presence of IL-1β, IL-1RN, and TNF-α variants was not associated with an increased risk of upper gastrointestinal bleeding.

## In Brief

Forgerini et al. investigated the role of seven genetic variants in the risk of upper gastrointestinal bleeding as an adverse drug reaction. In 289 participants (50 cases and 189 controls), the presence of seven variants in the IL-1β, IL-1RN, and TNF-α genes was not associated with susceptibility to acetylsalicylic acid-induced upper gastrointestinal bleeding.

## Highlights

■ This study contributes data on the pharmacogenomics of acetylsalicylic acid.

■ Brazilian data contribute to reducing the lack of diversity in genetic studies examining unrepresented groups.

■ The genetic variants evaluated were not associated with gastrointestinal bleeding.

## Graphical abstract

**Figure:**
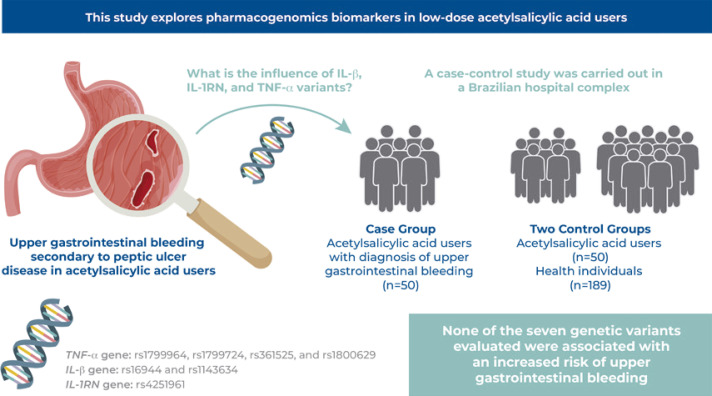

## INTRODUCTION

Upper gastrointestinal bleeding and perforated gastroduodenal ulcers are among the most serious complications of therapy with non-steroidal anti-inflammatory drugs (NSAIDs), including acetylsalicylic acid in low doses,^(1)^ and may lead to hospitalization, death, and increased healthcare costs.^(2)^

Understanding the molecular mechanisms involved in upper gastrointestinal bleeding as an idiosyncratic response is scarce,^(3,4)^ and genetic predisposition has been studied to elucidate the variability in gastrointestinal complications experienced by patients in response to certain medications.^(5,6)^

The identification of genetic biomarkers that influence the understanding of inter-individual responses to the use of medicines is a focus of pharmacogenetics and genomics since different responses are often related to genetic variations.^(7)^ This pursuit aims to identify potential predictive markers and individualize the pharmacotherapy, enhancing effectiveness while minimizing associated risks, such as adverse drug events.^(8)^ Among the genetic variants, single nucleotide polymorphisms (SNPs) stand out, as they are frequent in the population (>1%) and are considered diagnostic and pharmacotherapeutic biomarkers.^(9)^

In this regard, the association between SNPs in genes involved in drug metabolism, platelet aggregation, and physiological functions in the gastric mucosa and increased risk of upper gastrointestinal bleeding is well established (*e.g*., *CYP2C9* and *PTGS1* genes).^(5,6,10,11)^ In contrast, evidence linking variations in inflammation-related genes to an increased rate of acetylsalicylic acid-induced adverse events is limited.

Tumor necrosis factor α (TNF-α) and interleukins 1-β and 1-RN (IL-β and IL-1RN) are pro-inflammatory cytokines related to the inflammatory response in the pathogenesis of peptic ulcer disease and involved in a variety of biological activities, including platelet aggregation.^(12)^ SNPs in these genes have been associated with the development of several conditions, such as cardiovascular disease and gastric cancer,^(13,14)^ in addition to seeming to be related to acetylsalicylic acid-induced complications, such as urticarial or asthma.^(15,16)^ However, data on gastrointestinal disorders are contradictory and limited.^(17-19)^For some SNPs, the association data were not significant or were not possible to analyze due to the small sample size of previous studies.^(6,18)^

Considering that genetic factors seem to be one of the most important causes of acetylsalicylic acid-induced upper gastrointestinal bleeding, investigating the role of these SNPs in the Brazilian population would aid in reducing the lack of diversity in human genetic studies from underrepresented groups and contribute to data on individuals mostly likely to benefit from their use to prevent cardiovascular events.^(20)^ In addition, admixture populations, such as the Brazilian population, represent ideal subjects for pharmacogenetic and genomics studies. Here, polymorphic loci and linkage disequilibrium can be used to infer the genetic basis of response to pharmacotherapy.^(21)^

## OBJECTIVE

Therefore, we aimed to investigate the role of seven variants in the *TNF-*α, *IL-*β*,* and *IL-1RN* genes in association with the risk of upper gastrointestinal bleeding in users of low-dose acetylsalicylic acid for the prevention of cardiovascular events.

## METHODS

### Ethical approval

This study was approved by the Research Ethics Committees of the *Hospital das Clínicas* of the *Faculdade de Medicina de Ribeirão Preto* of the *Universidade de São Paulo* (USP) (CAAE: 53753115.4.0000.5440; #1.536.886) and the *Universidade Estadual Paulista “Júlio de Mesquita Filho*” (UNESP) (CAAE: 53753115.4.3001.5426; # 1.657.615).

### Study design and setting

A case-control study was carried out in the hospital complex of the *Hospital das Clínicas* of the *Faculdade de Medicina de Ribeirão Preto* (HCFMRP-USP), Brazil, which serves 49 municipalities and approximately 2,461,143 inhabitants.

This study complied with the Strengthening the Reporting of Observational Studies in Epidemiology (STROBE) guidelines.^(22)^

### Participants

#### Case Group

The Case Group consisted of patients diagnosed with upper gastrointestinal bleeding through upper digestive endoscopy or surgical procedure (laparoscopy) who were administered a low dose of acetylsalicylic acid.

Upper gastrointestinal bleeding was defined as the presence of an ulcer, gastric or duodenal lesions, with or without recent bleeding, and associated with signs and symptoms of hematemesis, melena, hematochezia, intense sweating, dizziness, abdominal pain, nausea, and vomiting.

The following subjects were included in the study: patients older than 18 years admitted to the hospital complex with clinical signs and symptoms of upper gastrointestinal bleeding; patients without signs and symptoms but with findings of recent bleeding on upper digestive endoscopy (active bleeding, adherent clot, and visible vessel); and patients admitted with symptoms of perforating acute abdomen confirmed by imaging tests (X-ray or CT scan).

The following exclusion criteria were applied: (i) individuals who were diagnosed with upper gastrointestinal bleeding but were not administered low-dose acetylsalicylic acid; (ii) those who were hemodynamically unstable, intubated, or died; (iii) those admitted with gastrointestinal bleeding related to the presence of esophageal varices, gastric cancer, *Mallory-Weiss* syndrome, cirrhosis, portal hypertension, and/or Dieulafoy lesions; (iv) those subjected to upper digestive endoscopy after 48 hours of hospitalization; (v) in-hospital upper gastrointestinal bleeding; (vi) hospitalization in 15 days prior to the current hospitalization; and (vii) patients unavailable for interview before hospital discharge.

The assessment of the endoscopy reports was performed daily by three previously trained researchers (two physicians and one pharmacist).

## Control Group

Two Control Groups were defined: one group comprised patients consuming a low dose of acetylsalicylic acid (100mg) for the prevention of cardiovascular events without the signs of upper gastrointestinal bleeding at the time of the interview and another group of healthy controls (nonusers of low-dose acetylsalicylic acid).

The Control Groups were recruited as follows:

1) Patients admitted to the hospital complex upon follow-up with the cardiologist (Control Group of patients administered a low dose of acetylsalicylic acid); 2) Patients scheduled for non-painful mild surgeries in the hospital complex, unrelated to the use of NSAIDs or low-dose acetylsalicylic acid and gastrointestinal disorders, such as lipoma, prostatic adenoma or hyperplasia, eye cataract, hernia, and septoplasty (Control Group of healthy subjects).

Controls were matched by sex, age (±5 years), and recruitment date (± 3 months). For each case, a low-dose acetylsalicylic acid user from the Control Group and up to three healthy controls were matched.

Upper digestive endoscopy was not performed in Control Group participants due to ethical considerations.

The exclusion criteria for the Case and Control Groups were as follows: participants with a history of neoplasms and coagulopathies, those who were HIV-positive, narcotics, carriers of nasogastric or percutaneous tubes, and those who did not reside in the study region for at least three months.^(10)^ All participants were unrelated biologically.

## Data collection

Participants were recruited through face-to-face interviews during the period from June 2016 to March 2020. The interviews were conducted by four previously trained researchers (two pharmacists and two physicians) through a questionnaire previously designed for this study^(10)^ and translated and adapted for Brazil.

The patient and/or family member provided answers on the following variables: sex, age, self-declared ethnicity, schooling, body mass index, personal history of gastrointestinal diseases, comorbidities, pharmacotherapy in use, and consumption of tobacco, alcohol, and coffee.

After the interview, venous blood was collected from all participants in EDTA tubes (5.0mL).

## Pharmacotherapy in use

An index date was considered, marked as the onset signs and symptoms of upper gastrointestinal bleeding for cases and as the date of interview for the controls.^(7,20)^ To assess an association between the pharmacotherapy in use and the risk of upper gastrointestinal bleeding, an etiological window of seven days from the index date was considered.^(6,10,23,24)^

Participants recalled chronic medication usage and self-medication in the two months prior to the index date during the interview. An anamnesis of the pharmacotherapy was carried out for all participants, considering the name of the medicine, Anatomical Therapeutic Chemical Code (ATC), indication of use, pharmaceutical form, dosage, and duration of use.

## Alcohol and coffee consumption and smoking

Alcohol and coffee consumption and smoking were evaluated due to their potential influence on damage to the gastrointestinal tract, as they can alter the balance between protective defense mechanisms and aggressive factors.^(25)^

Alcohol intake was calculated based on the estimated average glass volume and ethanol content for each type of alcoholic beverage and quantity and frequency of consumption (drinks per day, week, and month). To calculate the amount of alcohol consumed in grams, the content per 100mL of each fermented alcoholic beverage was defined as 11g for wine and 4.5g for beer, and per 50mL of liquors and distillates beverage (whisky, cachaça, and vodka), which was set at 20g. Alcohol intake was stratified into 0g of alcohol (abstainers), >0 to ≤25g of alcohol/week, >25 to ≤50g of alcohol/week, and >50g of alcohol/week. This stratification was based on the proposal by Wood and colleagues (2018), who investigated the risks associated with the amount of alcohol consumed weekly by 599,912 current drinkers.^(26)^

Coffee intake was recorded according to the amount and frequency of consumption (cups per day, week, and month). The volume of the cup was defined as 100mL and the coffee consumption was stratified into no consumption (0mL), >0mL ≤100mL, >100mL ≤300mL, and >300mL.

Tobacco smoking was stratified according to the number of cigarettes consumed per day: 0 cigarette (non-smokers and ex-smokers), 1 to 15 cigarettes/day, and >15 cigarettes/day.^(10)^

## *Helicobacter pylori* serology

The presence of IgG antibodies to *Helicobacter pylori* infection was determined in the human serum via the chemiluminescence technique. Participants samples were analyzed at the *Núcleo de Atendimento à Comunidade* (NAC) of the *Faculdade de Ciências Farmacêuticas* at UNESP and Álvaro Apoio, an accredited laboratory.

## DNA extraction, selection of genetic variants, and genotyping

Genomic DNA was extracted from venous blood samples [Maxwell^®^ 16 Blood DNA Purification Kit (Promega, Madison, WI, USA)]. DNA concentration and purity were determined by a fluorescence technique [Invitrogen Qubit 4 Fluorometer and the Qubit™ dsDNA HS Assay kit (Applied Biosystems, Foster City, USA)].

The following SNPs were selected according to their potential relevance in gastrointestinal disorders (ulcer or bleeding):^(18,27,28)^one in the promoter region of the *IL-*β gene [rs16944, A > G (Assay C_1839943_10)] and one in the exon 5 [rs1143634, G > A (Assay C_9546517_10)]; one in the promoter region of *IL-1RN* gene [rs4251961, G > A (Assay C_32060323_20)]; and four located in the promoter region of the *TNF-*α gene [rs1799964, T > C (Assay C_7514871_10); rs1799724, C > T (Assay C_11918223_10); rs361525, G > A (Assay C_2215707_10) and rs1800629, G > A (Assay C_7514879_10)].

The rsID number of SNPs was confirmed in dbSNP of the National Library of Medicine (https://www.ncbi.nlm.nih.gov/snp/), and the clinical relevance of each variant was verified in the literature. Single nucleotide polymorphisms in promoter regions of the *IL-*β, *IL-1RN*, and *TNF-*α genes were selected due to reports of potentially altered rates of gene transcription and cytokines released.^(29,30)^rs1143634 (*IL-*β gene), also known as +3954C>T, is a silent coding sequence variant, and this SNP might increase the release of IL-1β.^(31)^

Analyses of SNPs were performed at the Laboratory of Molecular and Microorganism Biology of *Universidade Estadual Paulista* “*Júlio de Mesquita Filho*” (UNESP) in São Paulo via real-time polymerase chain reaction technique (7500 real-time equipment). The following cycling conditions were used: 60^o^C for 1 minutes, 95^o^C for 10 minutes, 40 cycles of 95^o^C for 15 s and 60^o^C for 1 minute, and 60^o^C for 1 minute.

For internal quality control, 10% of the DNA samples were randomly selected for repeat genotyping. Allelic discrimination plots were analyzed in the Data Connect cloud from Applied Biosystems Thermo Fisher Scientific (https://apps.thermofisher.com/apps/spa/#/dataconnect).

## Study size

The sample size was calculated based on the frequency of the rs4251961 in the Brazilian population (ABraOM database, https://abraom.ib.usp.br/) and the odds ratio (OR) of the presence of this variant in the risk of upper gastrointestinal bleeding in users of low-dose acetylsalicylic acid (data were derived from the Spanish population due to lack of data in the Brazilian population).^(10)^

A calculator with the following variables was used: prevalence of 32% for the rs4251961.A (variant allele); 1% significance level; statistical power of 80%, and an OR of 4 (https://shiny.vet.unimelb.edu.au/epi/sample.size.mccs/). Therefore, the minimum sample size for this study was 42 cases and 42 controls.

## Statistical analysis

The allelic and genotypic frequencies of the evaluated SNPs were presented according to the three groups of study (Case and Control Groups). Deviations from the *Hardy-Weinberg* Equilibrium were verified by the χ^2^ test.

Continuous variables were analyzed by Student’s *t*-test, and categorical variables were analyzed by χ^2^ test or Fisher Exact test, when appropriate. Comparison of the frequencies of variables, genotypes, and minor allele frequency (MAF) between the groups was analyzed by χ^2^ test.

The risk of upper gastrointestinal bleeding was estimated by OR with a 95% confidence interval (95%CI) using unconditional logistic regression models. For the selection of confounding variables for the unconditional logistic regression models, a bivariate analysis was performed with each of the variables in relation to the outcome variable (upper gastrointestinal bleeding).

Unconditional logistic regression models were constructed with genetic variants, and all predictor variables that met the p≤0.20 criterion. Genetic variants were categorized into homozygous for the wild-type allele, heterozygous, and homozygous for the variant allele. To increase the statistical power of the analyses, genetic variants were also categorized into homozygous for the wild-type allele and ‘genetic variation’, which consists of participants carrying the variant allele (heterozygous and homozygous for the variant allele). The reference category of the genetic variants was defined as homozygous for wild-type allele.

Statistical analyses were carried out using the SPSS 26.0 software (IBM Company, Chicago, IL, United States).

## Data transparency and accessibility

Supplementary materials are available in the Open Science Framework (doi: 10.17605/OSF.IO/4SG93).

## RESULTS

### Study population

We evaluated 2,883 reports of upper digestive endoscopy (2,557 patients) from June 2016 to March 2020, and 50 low-dose acetylsalicylic acid users were diagnosed with upper gastrointestinal bleeding. Matched by sex, age, and time of hospital admission, 50 low-dose acetylsalicylic acid users without gastrointestinal complaints were recruited (Control Group of acetylsalicylic acid users). Matched by sex, age, and date of recruitment, a second group of controls, composed of 189 healthy individuals, was also recruited.

In both the Case and Control Groups, most participants were male, older individuals, self-identified as White, non-smokers, abstainers from alcohol, and reported daily consumption of coffee. The Case Group had a higher frequency of participants diagnosed with cardiovascular disease (74.0% *versus* 54.0% and 18.5%) and *Helicobacter pylori* infection (76.0% *versus* 54.0% and 59.8%), and those who consumed oral anticoagulants (18.0% *versus* 2.0% and 5.3%) and NSAIDs (14.0% *versus* 8.0% and 7.4%) when compared to both Control Groups (low-dose acetylsalicylic acid users and healthy controls) (Table 1).

**Table 1:** Baseline description of the participants in the Case and Control Groups (low-dose acetylsalicylic acid users and healthy controls)

	Case Group n=50 (%)	Control Group of low-dose ASA users n=50 (%)	p value^†^	Healthy Control Group n=189 (%)	p value^‡^
Demographic variables					
Sex (male)	36 (72.0)	35 (70.0)	0.826	139 (73.5)	0.826
Age [mean (±SD)]	69.4 (10.9)	69.0 (10.7)	0.373	67.7 (10.8)	0.153
Race (self-declared)					
White	39 (78.0)	38 (76.0)	0.459	139 (73.5)	0.895
Mixed (“*Pardo*” in Brazilian Portuguese)	5 (10.0)	8 (16.0)		26 (13.8)	
Black	6 (12.0)	3 (6.0)		22 (11.6)	
Asian	0	0		2 (1.1)	
Body mass index kg/m^2^					
Underweight (<18)	2 (4.0)	1 (2.0)	0.520	6 (3.2)	0.908
Normal (≥18 - ≤24)	15 (30.0)	10 (20.0)		51 (27.0)	
Overweight (≥25 - ≤29.9)	20 (40.0)	18 (36.0)		71 (37.5)	
Obesity (≥30)	13 (26.0)	21 (42.0)		61 (32.3)	
Personal history of gastrointestinal diseases					
*Helicobacter pylori* infection	38 (76.0)	27 (54.0)	0.021^‡^	113 (59.8)	0.035^#^
History of ulcer	6 (12.0)	9 (18.0)	0.401	22 (11.6)	0.944
History of bleeding	4 (8.0)	9 (18.0)	0.234	23 (12.2)	0.615
History of dyspepsia	10 (20.0)	14 (28.0)	0.349	71 (37.6)	0.020^#^
Comorbidity					
Cardiovascular disease	37 (74.0)	27 (54.0)	0.037^‡^	35 (18.5)	<0.001^#^
High blood pressure	44 (88.0)	45 (90.0)	0.749	110 (58.2)	<0.001^#^
*Diabetes mellitus*	15 (30.0)	26 (52.0)	0.025^‡^	48 (25.4)	0.511
Dyslipidemia	13 (26.0)	37 (74.0)	<0.001^‡^	43 (22.7)	0.630
Drug therapy in use (ATC)					
Other antiplatelet agents (B01AC)	22 (44.4)	21 (45.7)	0.840	8 (4.2)	<0.001^#^
Proton pump inhibitors (A02BC)	11 (22.0)	16 (32.0)	0.007^‡^	34 (18.0)	0.510
Oral anticoagulants (B01A)	9 (18.0)	1 (2.0)	0.016^‡^	10 (5.3)	0.007^#^
NSAIDs (M01A)	7 (14.0)	4 (8.0)	0.340	14 (7.4)	0.142^#^
Tobacco consumption (number of cigarettes per day)					
0 cigarette (non-smoker/ex-smoker)	41 (82.0)	44 (88.0)	0.585	161 (85.2)	0.500
1 to 15 cigarettes	3 (6.0)	4 (8.0)		11 (5.8)	
>15 cigarettes	5 (10.0)	2 (4.0)		10 (5.3)	
Missing data	1 (2.0)	0		7 (3.7)	
Alcohol intake (mean grams of alcohol per week)					
0 gram (abstainer)	34 (68.0)	31 (62.0)	0.712	113 (59.8)	0.644
0 >grams ≤25	6 (12.0)	12 (24.0)		45 (23.8)	
25 >grams ≤50	4 (8.0)	5 (10.0)		13 (6.9)	
>50 grams	6 (12.0)	2 (4.0)		18 (9.5)	
Coffee intake (mean amount of coffee per day)					
mL = 0	3 (6.0)	4 (8.0)	0.585	12 (6.3)	0.312
>0 mL ≤ 100	29 (58.0)	33 (66.0)		132 (69.8)	
>100 > mL ≤ 300	8 (16.0)	8 (16.0)		23 (12.2)	
>300 mL	10 (20.0)	5 (10.0)		22 (11.7)	

p value is polychotomous and represents the entire variable.

^†^ Comparison of the frequency of variables between the Case Group and the Control Group of low-dose acetylsalicylic acid users using χ^2^ test or Fisher test, when appropriate; ^‡^ Comparison of the frequency of variables between the Case Group and healthy Control Group using χ^2^ test or Fisher test, when appropriate; ^#^ variables with p≤0.20 and selected for the unconditional regression models.

ASA: acetylsalicylic acid; ATC: Anatomical Therapeutic Chemical; NSAIDs: Non-steroidal anti-inflammatory drugs; SD: standard deviation.

### Genetic variants

All 289 individuals (50 low-dose acetylsalicylic acid users with upper gastrointestinal bleeding, 50 low-dose acetylsalicylic acid users of the Control Group, and 189 healthy controls) included in this study were successfully genotyped, and all variants met the criteria for the *Hardy-Weinberg* Equilibrium. The genotyping reproducibility obtained was 98%.

No significant differences were observed in the genotypes and allele frequencies of the TNF-α, IL-β, and IL-1RN variants between the Case Group (upper gastrointestinal bleeding) and the Control Group of low-dose acetylsalicylic acid users. The frequency of carriers of rs1800629.A was higher in the Case Group (upper gastrointestinal bleeding) than in the healthy Control Group (p=0.003) (Table 2).

**Table 2:** Frequency of *TNF-*α*, IL-*1β*,* and *IL-1RN* genotypes among participants in the Case and Control Groups (low-dose acetylsalicylic acid users and healthy controls)

	Case Group n=50 (%)	MAF	Control Group of low-dose ASA users n=50 (%)	MAF	p value^†^	Healthy Control Group n=189 (%)	MAF	p value^‡^
*TNF-*α gene								
rs1799964 (T > C)								
TT (reference homozygous)	26 (52.0)	0.30	33 (66.0)	0.19	0.212	93 (49.2)	0.31	0.893
TC (heterozygous)	18 (36.0)		15 (30.0)			75 (39.7)		
CC (variant homozygous)	6 (12.0)		2 (4.0)			21 (11.1)		
HWE	0.3124		0.8578			0.3248		
rs1799724 (C > T)								
CC (reference homozygous)	40 (80.0)	0.11	39 (78.0)	0.13	0.841	146 (77.2)	0.13	0.871
CT (heterozygous)	9 (18.0)		9 (18.0)			37 (19.6)		
TT (variant homozygous)	1 (2.0)		2 (4.0)			6 (3.2)		
HWE	0.5683		0.1487			0.0687		
rs361525 (G >A)								
GG (reference homozygous)	46 (92.0)	0.04	47 (94.0)	0.03	0.695	166 (87.8)	0.06	0.441
GA (heterozygous)	4 (8.0)		3 (6.0)			22 (11.6)		
AA (variant homozygous)	0		0			1 (0.6)		
HWE	0.7683		0.8269			0.7708		
rs1800629 (G >A)								
GG (reference homozygous)	43 (86.0)	0.07	39 (78.0)	0.12	0.436	183 (96.8)	0.02	0.003*
GA (heterozygous)	7 (14.0)		10 (20.0)			6 (3.2)		
AA (variant homozygous)	0		1 (2.0)			0		
HWE	0.5946		0.7077			0.8245		
*IL-1*β gene								
rs16944 (A >G)								
AA (reference homozygous)	15 (30.0)	0.44	17 (34.0)	0.37	0.551	113 (59.8)	0.24	<0.001*
AG (heterozygous)	26 (52.0)		23 (46.0)			59 (31.2)		
GG (variant homozygous)	9 (18.0)		5 (10.0)			15 (7.9)		
Missing data	0		5 (10.0)			2 (1.1)		
HWE	0.6963		0.5003			0.0753		
rs1143634 (A >G)								
AA (reference homozygous)	36 (72.0)	0.17	30 (60.0)	0.24	0.595	124 (65.6)	0.20	0.648
AG (heterozygous)	11 (22.0)		14 (28.0)			54 (28.6)		
GG (variant homozygous)	3 (6.0)		5 (10.0)			11 (5.8)		
HWE	0.1191		0.1113			0.1282		
*IL-1RN gene*								
rs4251961 (G >A)								
GG (reference homozygous)	19 (38.0)	0.36	18 (36.0)	0.39	0.827	77 (40.7)	0.37	0.488
GA (heterozygous)	26 (52.0)		25 (50.0)			83 (43.9)		
AA (variant homozygous)	5 (10.0)		7 (14.0)			29 (15.4)		
HWE	0.3636		0.7191			0.4006		

^*^ Statistical significance (p<0.05); ^†^ Comparison of genotypic frequencies of variants between the Case Group and the Control Group of low-dose acetylsalicylic acid users using χ^2^ test or Fisher test, when appropriate; ^‡^ Comparison of genotypic frequencies of variants between the Case Group and healthy Control Group using χ^2^ test or Fisher test, when appropriate.

ASA: acetylsalicylic acid; HWE: *Hardy-Weinberg* Equilibrium; MAF: minor allele frequency.

### Risk of upper gastrointestinal bleeding

To investigate possible associations between specific genotypes of the target genes and the risk of upper gastrointestinal bleeding, logistic regression models were obtained comparing the Case Group and Control Group of low-dose acetylsalicylic acid users, as well as the Case Group and healthy controls. None of the evaluated SNPs were associated with the risk of upper gastrointestinal bleeding (Table 3).

**Table 3:** Risk for upper gastrointestinal bleeding associated with TNF-α, IL-1β, and IL-1RN genotypes among participants in the Case and Control Groups (low-dose acetylsalicylic acid users and healthy controls)

	(Case Group/Control Group of low-dose ASA users)	OR^†^	95%CI^†^	p value^†^	(Case Group/ Control Group of healthy individuals)	OR^‡^	95%CI^**‡**^	p value^‡^
*TNF-*α gene								
rs1799964 (T > C)								
TT (reference homozygous)	26/33	1.000	-	-	26/93	1.000	-	-
TC (heterozygous)	18/15	1.393	0.45 – 4.31	0.565	18/75	0.966	0.34 – 2.73	0.948
CC (variant homozygous)	6/2	2.027	0.27 – 15.21	0.492	6/21	1.167	0.27 – 4.91	0.833
TC + CC	24/17	1.478	0.50 – 4.35	0.478	24/96	0.746	0.29 – 1.93	0.546
rs1799724 (C > T)								
CC (reference homozygous)	40/39	1.000	-	-	40/146	1.000	-	-
CT (heterozygous)	9/9	1.343	0.36 – 4.93	0.492	9/37	0.790	0.23 – 2.68	0.705
TT (variant homozygous)	1/2	1.824	0.16 – 20.48	0.896	1/6	2.172	0.21 – 22.80	0.518
CT + TT	10/11	0.713	0.20 – 2.48	0.595	10/43	0.921	0.30 – 2.87	0.887
rs361525 (G > A)								
GG (reference homozygous)	46/47	1.000	-	-	46/166	1.000	-	-
GA (heterozygous)	4/3	1.341	0.13 – 13.68	0.804	4/22	1.481	0.33 – 6.63	0.607
AA (variant homozygous)^#^	0/0	-	-	-	0/1	-	-	-
GA + AA	4/3	1.341	0.13 – 13.68	0.804	4/23	0.760	0.15 – 3.90	0.743
rs1800629 (G > A)								
GG (reference homozygous)	43/39	1.000	-	-	43/183	1.000	-	-
GA (heterozygous)	7/10	0.553	0.11 – 2.68	0.462	7/6	1.714	0.21 – 13.60	0.610
AA (variant homozygous)^#^	0/1	-	-	-	0/0	-	-	-
GA + AA	7/11	0.539	0.11 – 2.57	0.439	7/6	4.049	0.52 – 31.30	0.180
*IL-1B* gene								
rs16944 (A > G)								
AA (reference homozygous)	15/17	1.000	-	-	15/113	1.000	-	-
AG (heterozygous)	26/23	0.870	1.26 – 2.96	0.824	26/59	5.217	1.44 – 16.17	0.005*
GG (variant homozygous)	9/5	2.118	0.37 – 12.26	0.402	9/15	5.241	1.08 – 25.37	0.040*
AG + GG	35/28	1.031	0.32 – 3.35	0.960	35/74	5.223	1.75 – 15.62	0.003*
rs1143634 (A > G)								
AA (reference homozygous)	36/30	1.000	-	-	36/124	1.000	-	-
AG (heterozygous)	11/14	0.559	0.15 – 2.01	0.374	11/54	1.127	0.38 – 3.34	0.829
GG (variant homozygous)	3/5	0.988	0.10 – 9.35	0.991	3/11	1.589	0.27 – 9.27	0.607
AG + GG	14/19	0.620	0.19 – 2.06	0.435	14/65	1.490	0.55 – 4.05	0.435
*IL-1RN* gene								
rs4251961 (G > A)								
GG (reference homozygous)	19/18	1.000	-	-	19/77	1.000	-	-
GA (heterozygous)	26/25	0.710	0.23 – 2.17	0.549	26/83	1.179	0.43 – 3.20	0.747
AA (variant homozygous)	5/7	0.334	0.06 – 1.87	0.213	5/29	1.141	0.29 – 4.52	0.851
GA + AA	31/32	0.607	0.21 – 1.76	0.359	31/112	1.319	0.52 – 3.38	0.564

^†^ Analysis adjusted for the following confounding variables: *Helicobacter pylori* infection; cardiovascular disease; dyslipidemia; *diabetes mellitus*; use of oral anticoagulants and proton pump inhibitors; ^‡^ Analysis adjusted for the following confounding variables: *Helicobacter pylori* infection; personal history of dyspepsia; cardiovascular disease; high blood pressure; use of oral anticoagulants, non-steroidal anti-inflammatory drugs, and other antiplatelet agents; ^#^ It was not possible to evaluate the category of the variant homozygous genotype of rs361525 and rs1800269 due to the absence of participants in the Case Group.

ASA: acetylsalicylic acid; 95%CI: 95% confidence interval; OR: odds ratio.

## DISCUSSION

Although some inflammatory cytokines are known to be associated with gastritis, ulcer, and gastric cancer,^(32-35)^ few studies have examined the relationship between variants in genes encoding inflammatory mediators and acetylsalicylic acid-induced upper gastrointestinal bleeding. Hence, to examine the genetic predisposition of an individual to develop upper gastrointestinal bleeding, we evaluated the influence of seven SNPs in the *IL-*β (rs16944 and 1143634), *IL-1RN* (rs4251961), and *TNF-*α genes (rs1799964, rs1799724, rs361525, and rs1800629) in low-dose acetylsalicylic acid users, alongside a group of healthy controls, representative of the general population.

The expression level and concentration of IL-1β may be affected by rs16944 and rs1143634 variants.^(36)^ Changes in IL-1β expression can directly alter its transcription and increase its production in the gastric mucosa, which may result in greater suppression of gastric acid secretion, increased inflammation and, consequently, lesions.^(37)^ However, despite the report of an increased risk of peptic ulcer in users of low-dose acetylsalicylic acid (100mg) carrying the rs16944.G and rs1143634.G alleles in a Korean ethnic group (n=48),^(28)^ the presence of these SNPs was not associated with the risk of upper gastrointestinal bleeding in our study.

Moreover, individuals carrying rs16944.G and rs1143634.G are at an increased risk of inflammation-mediated diseases,^(38-41)^ requiring low-dose acetylsalicylic acid therapy. In our investigation, patients who consumed low-dose acetylsalicylic acid had a higher frequency of cardiovascular disease and diseases related to the metabolic syndrome - conditions often accompanied by chronic low-grade inflammatory states and increased serum concentration of pro-inflammatory cytokines,^(42)^ but no association was identified.

In contrast, a study performed with a Chinese population identified a reduced risk of gastric damage in the users of 100mg of acetylsalicylic acid carrying the rs16944.G allele.^(18)^ However, it is worth noting that the study conducted by Wu and colleagues (2016) presents some methodological concerns (*e.g*., unclear recruitment of participants and adjustment of statistical analyses for confounding variables) and did not present association data.^(18)^

One of the biological roles of IL-1β is to enhance the inflammatory response and induce the expression of other pro-inflammatory cytokine genes, such as *TNF-*α.^(43)^Nonetheless, despite reports that patients with gastrointestinal bleeding may have upregulated cytokines due to variants in the *TNF-*α gene,^(19,44)^ none of the four SNPs evaluated in this gene were associated with the risk of upper gastrointestinal bleeding.

Our findings coincide with Wang et al.^(19)^ who showed that rs361525 and rs1799724 (*TNF-*α gene) were not associated with the risk of upper gastrointestinal bleeding in users of low-dose acetylsalicylic acid (100mg) from China.^(18,19)^ In contrast, rs1799964 (*TNF-*α gene) was reported as a risk factor for upper gastrointestinal bleeding in a subgroup analysis of European descendants living in Spain (24 cases and 17 controls), since the recruitment of the participants was independent of the use of low-dose acetylsalicylic acid.^(6)^ Regarding rs1800269, no report is available in the literature about its influence on gastrointestinal complications in users of low-dose acetylsalicylic acid, precluding the comparison of our findings.^(27)^

Ultimately, although the interleukin-1 receptor antagonist, encoded by the *IL-1RN* gene, also plays a key role in modulating the inflammatory response in the gastrointestinal mucosa,^(45)^ our data did not indicate an association between the presence of the variant allele of rs4251961 and the risk of upper gastrointestinal bleeding in low-dose acetylsalicylic acid users. In line with this finding, the study conducted by Cho et al. suggested that this SNP was not associated with the development of peptic ulcers, one of the main causes of upper gastrointestinal bleeding.^(28)^

A possible rationale for the lack of risk of upper gastrointestinal bleeding in the presence of the seven SNPs evaluated in our study is that low-dose acetylsalicylic acid can attenuate acute inflammatory responses by increasing 15-epi-lipoxin A4, an endogenous protective lipid that plays a key role in regulating the inflammatory response and maintaining a healthy cardiovascular system.^(42)^ Remarkably, patients consuming low-dose acetylsalicylic acid presented a reduction in the levels of inflammatory cytokines^(46)^and an increase in the prevention of cyclooxygenase-mediated cell activation and proliferation, which might reduce the release of cytokines into the blood by acetylating cyclooxygenase.^(47)^

In line with this hypothesis, low-dose acetylsalicylic acid may decrease the magnitude of the risk of upper gastrointestinal bleeding, as a “negative” modifier, due to a biological interaction between low-dose acetylsalicylic acid and the SNPs in the evaluated genes. However, to support this hypothesis, it would be necessary to include and evaluate individuals exposed and unexposed to low-dose acetylsalicylic acid in both study groups (Case and Control Groups).^(48)^

Finally, it is noteworthy that the previous studies mentioned above evaluated the influence of SNPs related to inflammatory cytokines and gastrointestinal complications of low-dose acetylsalicylic acid use in Chinese,^(18,19)^ Korean,^(28)^ and European descendants.^(6)^ Considering the caveat of extrapolating data from populations of homogeneous ethnic groups,^(49,50)^ this study is groundbreaking for contributing data on the pharmacogenomics of acetylsalicylic acid, which is less explored in the admixture population.^(51)^ Therefore, the differences in ethnic structure between populations should be considered in genetic studies.

Limitations of this study include the small sample size, a common limitation in genetic studies,^(52)^ which also precluded combined analysis of IL-β, IL-1RN, and TNF-α variant alleles. Furthermore, we analyzed a limited number of SNPs (seven), predominantly located in the promoter region of the genes rather than the coding region, which may justify why the null hypothesis was not rejected. Therefore, our findings should be confirmed in larger studies, as these variants may have direct implications for the safety of chronic acetylsalicylic acid users.

The primary strengths of this study include the four-year screening of low-dose acetylsalicylic acid users with upper gastrointestinal bleeding, the recruitment of two Control Groups (low-dose acetylsalicylic acid users and healthy subjects), and the face-to-face data collection with the inclusion of several confounding variables for upper gastrointestinal bleeding.

## CONCLUSION

The presence of variant alleles of the *IL-*β, *IL-1RN,* and *TNF-*α genes was not associated with susceptibility to acetylsalicylic acid-induced upper gastrointestinal bleeding.
